# The Role of Subretinal Injection in Ophthalmic Surgery: Therapeutic Agent Delivery and Other Indications

**DOI:** 10.3390/ijms241310535

**Published:** 2023-06-23

**Authors:** Domenico Tripepi, Assad Jalil, Naseer Ally, Matilde Buzzi, George Moussa, Pierre-Raphaël Rothschild, Tommaso Rossi, Mariantonia Ferrara, Mario R. Romano

**Affiliations:** 1 Department of Biomedical Sciences, Humanitas University, 20090 Pieve Emanuele, Italy; 2 Manchester Royal Eye Hospital, Oxford Road, Manchester M13 9WL, UK; 3 Lancashire Teaching Hospitals NHS Foundation Trust, Preston PR2 9HT, UK; 4 Department of Ophthalmology, Hôpital Cochin, Assistance Publique-Hôpitaux de Paris, 75014 Paris, France; 5 Centre de Recherche des Cordeliers, INSERM, UMR_1138, Université Paris Cité, 75270 Paris, France; 6 IRCCS Fondazione Bietti-ONLUS, 00184 Rome, Italy; 7 Faculty of Medicine, University of Malaga, 29016 Malaga, Spain; 8 Eye Center, Humanitas Gavazzeni-Castelli, 24128 Bergamo, Italy

**Keywords:** drug delivery, full-thickness macular hole, gene therapy, submacular hemorrhage, subretinal fluid application, subretinal injection, tissue plasminogen activator

## Abstract

Subretinal injection is performed in vitreoretinal surgery with two main aims, namely, the subretinal delivery of therapeutic agents and subretinal injection of fluid to induce a controlled and localized macular detachment. The growing interest in this technique is mainly related to its suitability to deliver gene therapy in direct contact with target tissues. However, subretinal injection has been also used for the surgical management of submacular hemorrhage through the subretinal delivery of tissue plasminogen activator, and for the repair of full-thickness macular holes, in particular refractory ones. In the light of the increasing importance of this maneuver in vitreoretinal surgery as well as of the lack of a standardized surgical approach, we conducted a comprehensive overview on the current indications for subretinal injection, surgical technique with the available variations, and the potential complications.

## 1. Introduction

Subretinal injection is a delicate surgical maneuver whose application in vitreoretinal surgery is progressively increasing. This is mainly due to the suitability of subretinal injection to deliver a controlled amount of therapeutic agent directly to the retinal pigment epithelium (RPE) cells and photoreceptors. Additional advantages include the need for a reduced concentration of drug and the delivery in an immune-privileged and anatomically closed subretinal space [1,2]. Furthermore, recent evidence supports the safety of this technique reporting minimal iatrogenic trauma and potential functional recovery following the limited macular detachment induced by this surgical technique [3].

Based on this, subretinal injections are now the established preferred route of administration of gene therapy for the treatment of inherited retinal diseases (IRDs), where RPE cells and photoreceptors are primarily affected [2]. In terms of drug delivery, the subretinal injection of tissue plasminogen activator (tPA) is a well-established therapeutic option for the management of submacular hemorrhages (SMHs) of different etiologies [4]. Subretinal injection of bevacizumab and/or air can be used in the same context for large submacular bleeding [5]. Finally, subretinal fluid injection is used to treat macular folds by inducing a limited retinal detachment before unfolding the retina [6], and also gaining popularity as a surgical option for full-thickness macular holes (FTMHs) repair [7].

Despite the growing role of subretinal injection in ophthalmic surgery, there is no standardized surgical technique, and different variations are described in the currently available literature [8]. With this background, our review aims to summarize the current concepts on subretinal injection, including the indications, surgical technique with its variations, and the potential complications. A comprehensive literature review was performed by searching on PubMed and Embase. The following MeSH terms were used for the literature search: subretinal injection, subretinal gene therapy, subretinal fluid application, subretinal tissue plasminogen activator, and combinations of them. Only publications written in English were included. Retrospective and prospective studies with sample size of at least five eyes were included as well as authoritative reviews. The first screening of publications was performed by title and abstract, and only studies pertinent to this review were included.

## 2. Subretinal Injection for Gene Therapy

Inherited retinal diseases (IRDs) comprise a small proportion of all retinal diseases. They are a group of diseases with a multitude of phenotypic expressions, wherein the etiology is a genetic abnormality within the cells of the retina or choroid [9,10]. Since the defect is at a cellular level, an effective treatment is the delivery of a “normal” genetic payload into the retinal cells of affected patients [10]. This can be achieved using adeno-associated virus (AAV) or lentivirus vectors which transfect the gene(s) into the affected host cells [10,11]. These viral vectors have also shown a good safety profile, not eliciting a host immune response in animal models [12]. Table 1 summarizes the IRDs for which subretinal gene therapy is currently evaluated in clinical trials. In addition, ongoing trials are evaluating gene the efficacy of gene therapy also in other retinal diseases, such as age-related macular degeneration.

Subretinal injection is the preferred route of administration of gene therapy as it is superior to the other potential routes, which include delivery into the anterior chamber and vitreous cavity [8,9,11].

Indeed, the subretinal delivery of a therapeutic agent allows the direct delivery to the targeted disease cells with less overflow into other uninvolved sites, low immunogenicity, good biodistribution control, and the overcoming of limitations of other routes of administration, such as the poor penetration of the viral vector through the internal limiting membrane (ILM) in case of intravitreal injection [8,12,13].

Each patient should be assessed on an individual basis through a comprehensive assessment including clinical appearance and genotyping, in order to evaluate the suitability for the current available gene therapies.

### Surgical Technique

The patient may be pretreated with oral corticosteroids to reduce the immune response to viral vectors, which has been described with retinal gene therapy [14]. The steroid dose can vary, but usually involves starting prednisolone at 1 mg/kg a few days prior to surgery followed by a gradual taper over two to three weeks [15,16]. The surgery can be performed under local or general anesthesia.

The standard technique for subretinal injection of gene therapy involves carrying out three-port 23- or 25-gauge (G) vitrectomy (PPV). After inducing the posterior vitreous detachment (if needed) and completing the core and peripheral vitrectomy along with peripheral retina examination, it should be ensured that the injection site is completely clear of any residual vitreous. This maneuver can be facilitated by the use of diluted triamcinolone acetonide injected intravitreally to the site of interest.

**Figure 1:**
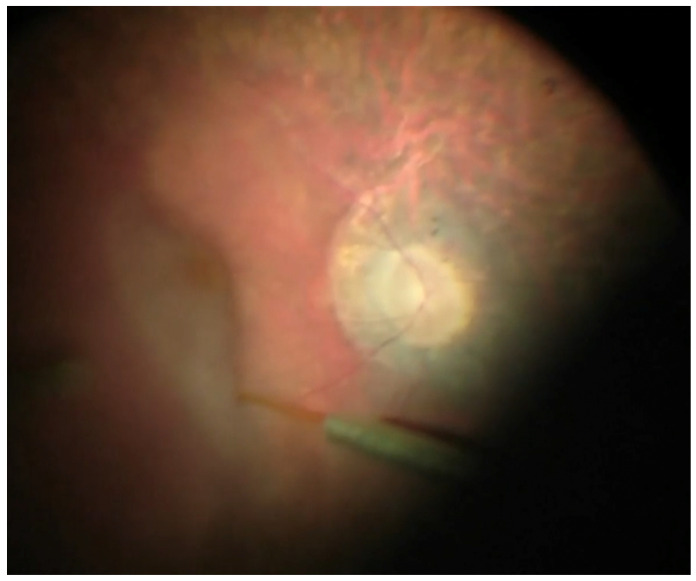
Subretinal gene therapy. Subretinal injection of gene therapy in a patient with retinitis pigmentosa and RPE-65 mutation. Injection site was placed along the supero-temporal vascular arcade. The injection, fovea-involving, was performed automatically, keeping a controlled injection pressure to avoid the overstretching of the retina and, in particular, of the fovea.

The site of gene therapy injection should be selected taking care to avoid areas of advanced retinal atrophy, epiretinal membranes (ERMs), blood vessels, and the papillomacular bundle [16]. If needed, thick ERMs can be removed before performing the injection [16]. The area within the temporal vascular arcades is commonly considered the preferred area for gene therapy (Figure 1) [15]. It is still controversial whether to directly detach the fovea with the injection as IRD-related foveal thinning and the potential risk of FTMH along with the difficulty to detach this area are potential factors against the subfoveal detachment [15,16].

There are different ways in which the subretinal injection can then be performed, namely, manually or automatically, and with or without formation of a pre-bleb [15,17] Automated subretinal injections are performed by controlling the injection pressure and infusion speed using the foot pedal of the vitrectomy machine. Manual injections rely on the surgeon or surgeon’s assistant to depress the plunger of the syringe [9,16,18].

**Figure 2:**
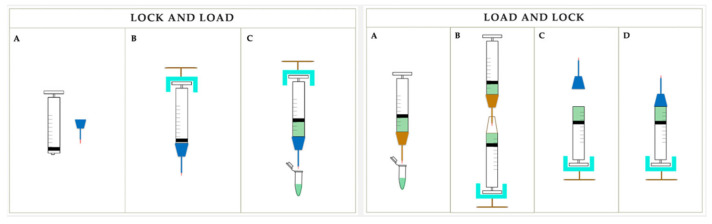
Schematic diagram of “lock and load” or a “load and lock” method to load the therapeutic agent. On the **left** “lock and load” (**A**) schematic illustration of viscous fluid injection mode (VFIM) syringe and subretinal cannula; (**B**) cannula and VFIM syringe for subretinal injection are assembled; (**C**) the vector solution is aspirated from the vial. On the **right** “load and lock” (**A**) the vector solution is aspirated using a separate syringe; (**B**) the vector is then transferred from the first syringe into the distal end of the VFIM syringe; (**C**) VFIM and subretinal cannula are assembled; (**D**) the system is ready for use.

Creation of a pre-bleb with balanced salt solution (BSS) prior to the subretinal injection of gene therapy vector has the potential benefit of conserving viral vector [15]. Indeed, in cases where a pre-bleb is not created, loss of viral vector can occur if a bleb does not form with subretinal injection of the genetic payload. However, it is worth noting that the pre-bleb size affects how much vector can be delivered into the subretinal space, as a larger pre-bleb can result in decreased injectable volume of vector while a smaller pre-bleb facilitates a larger delivery of viral vector [16]. This is due to overstretching of the retina, especially at the fovea, with increased risk of macular hole formation. In certain cases, however, a larger pre-bleb may be needed if the retina is more adherent to underlying structures, as in the case of choroideremia [16]. The volume injected into the subretinal space also differs according to the type of disease, with choroideremia requiring 100 µL and IRD caused by RPE65 mutation requiring up to 300 µL [19]. In preparation for pre-bleb creation, a 10 mL viscous fluid injection mode (VFIM) syringe is filled with BSS, taking care to avoid turbulence so as to avoid microbubbles. This can be achieved via two methods. Both BSS and viral vector can be loaded into the system via a “lock and load” or a “load and lock” method (Figure 2) [8]. In the former, the injection system is completely assembled and the viral vector/BSS is aspirated from a vessel into the system. The latter method uses a second syringe to inject the solution into the distal end of the VFIM syringe. Fischer et al. [8] showed that the “lock and load” system may be associated with the formation of less microbubbles compared to the “load and lock” system.

A 23- or 25-G cannula with a retractable cannula, with size variable from 38- to 41-G, is then attached to the VFIM syringe [15,16,20]. The retraction of the cannula results in increased stiffness and can be helpful to achieve penetration of the retina [16]. As per surgeon preference, the subretinal cannula can be beveled with scissors in order to be used bevel-up at the time of the injection [17]. However, the beveling of the cannula may be associated with a transient pseudo-schitic appearance during injection on OCT due to the hydration of the outer plexiform layers [17]. In the “lock and load” method this is performed prior to the aspiration of BSS/viral vector, whilst in the “load and lock” method it is performed after. The entire system is then flushed to ensure that there are no bubbles within the system. Once it has been flushed, if a pre-bleb with BSS is going to be created, additional system flushes to clear bubbles may be performed outside the eye in a container with BSS, and in the mid-vitreous cavity prior to injection. A new system specifically designed for micro-injection (less than 1 mL, ±1 psi control) equipped with cannulas for subretinal injections, has recently obtained the FDA clearance, becoming the first system available approved for subretinal injection (EVA INICIO, DORC, Zuidland, The Netherlands). If, despite the flushing, large air bubbles are created in the subretinal space, the injection needle can be used in extrusion mode to remove them and avoid the potential consequent volume issues [16].

The injection pressure is then set on the machine, more commonly between 12–18 psi [15,16]. Younger patients may require higher infusion pressures during creation of the pre-bleb [21]. The identification of the optimal injection pressure is crucial for the safety of this technique as both low and high injection pressure may be associated with mechanical RPE/retinal damage due to a prolonged permanence of the needle in the subretinal space and the high flow generated, respectively [22]. In this regard, a recent study on porcine eyes in vivo reported that injection pressure up to 24 psi might be safe, resulting in minimal retinal damage and no functional changes on the multifocal electroretinogram [22]. To note, the injection pressure to initiate the bleb formation is higher than that required for the bleb propagation [21]. The infusion pressure of the vitrectomy machine is lowered to 10 mmHg to allow for a smooth subretinal injection.

A useful adjunct during the process of subretinal injection can be the microscope-integrated optical coherence tomography (MI-OCT). The MI-OCT reticule can be positioned close to the injection site prior to insertion of the cannula. Once the injection begins, if the cannula is correctly placed, the immediate separation and elevation of the retina from RPE is seen. MI-OCT allows monitoring of successful bleb creation with the “fleur de lis” sign, with the central “stem” made by the subretinal cannula and the petals on either side made by the adjacent retina elevated by the subretinal bleb [20]. MI-OCT also ensures that overstretching of the retina, particularly of the fovea, is minimized. In this regard, inversion of the foveal contour is an indication that the fovea is overstretched [17].

If the gene vector is injected after the formation of the pre-bleb, the same retinotomy should be used for the vector injection. Three main complications can be associated with the creation of the pre-bleb and the subsequent gene therapy injection. In particular, a second retinotomy can be created accidentally or the first retinotomy can be widened. leading to an increased risk of reflux of the vector. This can potentially not only reduce the drug in the subretinal space where it is needed, but also increase the risk of immune reaction to the viral capsids escaping into the vitreous cavity. Finally, as mentioned above, the higher subretinal volume can increase the risk of retinal overstretching and, potentially, FTMH formation if the fovea is detached.

If, no pre-bleb has been created, the vector itself is used to detach the retina and create the bleb [16]. Sometimes more than one bleb is needed to treat larger contiguous and/or noncontiguous areas [15,21]. In cases of focal retinal thinning, heavy liquid can be used to provide internal tamponade of the retina and prevent retinal breaks [15,23]. The assistant surgeon can help monitoring the amount of vector injected [16].

After the drug has been delivered into the subretinal space, a vitreous cavity washout is performed to remove any vector that has refluxed into the vitreous cavity [15,16,21]. At this point, performing fluid–air exchange may assist in coalescing multiple blebs and spreading the area of effect of the genetic payload [15,16,21]. The use of air can also be used to push the subretinal fluid more posteriorly and detach the fovea if needed. Leaving the vitreous cavity air-filled, especially with larger volumes of 300 µL of the drug administered subretinally, can increase the risk of macular fold formation [24]. Some surgeons prefer to leave the vitreous cavity fluid-filled, to reduce this risk and to prevent viral vector reflux back into the vitreous cavity [19]. It has been suggested that sclerotomies should be sutured to prevent hypotony and reflux of vector out of the eye [19]. After the surgery, the patient is usually advised to lie on their back for 24 h to ensure that the drug stays in the macula till the bleb is absorbed, and to minimize the risk of macular fold.

Some limitations of subretinal injections include physiological surgeon tremor, and instrument drift. A potential future development was investigated by Ladha et al., who showed that robotic-assisted subretinal injection helps reduce the aforementioned surgeon-related factors when performing subretinal injections in artificial retina models [25]. Several aspects were suggested as advantages of telemanipulated robotic systems for subretinal injection, such as increased motion scaling, surgical automation, remote control option, and preset boundaries to movements [25]. Recent experimental studies reported advantaged in robot-assisted subretinal injection compared with the manual one, including improvement in tremor, drift, size of retinal hole, and injection time [26], as well as reduced fatigue and increased stability [27].

An alternative to the traditional subretinal injection technique following vitrectomy is being explored currently using an orbital subretinal delivery system (Gyroscope Therapeutics Orbit SDS™). In this technique, a flexible cannula mounted on a delivery system is introduced into the suprachoroidal space through a scleral incision and progressed towards the posterior pole using MI-OCT, before gently introducing the microneedle into the subretinal space [28]. This suprachoroidal approach to subretinal injection obviates the need for vitrectomy and retinotomy, and delivers the precise amount of drug with no potential loss into the vitreous cavity. Potential risks include suprachoroidal hemorrhage and development of subretinal neovascular membrane. Further studies are needed to establish the role of this technique in the subretinal delivery of drugs.

## 3. Subretinal Injection of tPA for Submacular Hemorrhage

Submacular hemorrhage is a harmful and sight-threatening complication associated with neovascular age-related macular degeneration and polypoidal choroidal vasculopathy, but, less commonly, it can also be associated with ocular trauma, pathological myopia, or rupture of retinal arterial microaneurysm [4]. The accumulation of blood in the subretinal space can damage the retina irreversibly through different mechanisms: the blood can act as a barrier impeding the diffusion of nutrients and waste products, the shrinking clot and the iron contained in the red blood cells can damage the photoreceptors, the former exerting traction, and the latter exerting a direct toxic effect [29,30]. Submacular hemorrhage can be associated with poor visual prognosis and a prompt treatment is necessary in an attempt to optimize the functional outcomes, as the damage degree increases as the persistence of the subretinal blood increases [4,31].

Several therapeutic options, and combinations of them, have been proposed for the management of submacular hemorrhage, including antivascular endothelial growth factor (VEGF) agents, photodynamic therapy, pneumatic displacement using expansile gases, intravitreal injection of tPA, and PPV. PPV in this context allows the performance of different maneuvers, such as subretinal injection of tPA, active drainage of subretinal blood, RPE patch grafting, macular translocation, or removal of the choroidal neovascularization [32,33].

The rationale of the subretinal injection of tPA relies on the thrombolytic activity of this serine protease which binds the fibrin, forming a complex able to activate the plasminogen to plasmin, and, thus, has the ability to lyse the blood clot. By liquefying the blood, tPA can not only facilitate its drainage/displacement but also reduce the risk of iatrogenic damage to photoreceptors during clot removal as well as the need for large retinotomies and the associated risk of retinal detachment, epiretinal membrane (ERM), and proliferative vitreoretinopathy [34].

### Surgical Technique

The use of subretinal tPA was first described by Peyman et al. [35] and involved a standard three-port PPV, the injection of the tPA into the submacular hemorrhage, and the creation of a small retinotomy to actively drain the liquefied subretinal blood 30–60 min after the injection. Over the years, the approach to the subretinal blood changed from the active drainage/removal, which can be associated with iatrogenic damage itself [35,36,37], to the pneumatic displacement of the liquefied blood, with the primary aim to mobilize it away from the fovea [4].

In addition, several surgical variants have been described and multiple aspects of this surgical technique are still not standardized, such as the site of injection, the dose of tPA, the need to drain the subretinal blood, and the use of intraoperative adjuvants.

Different sites have been proposed for the injection, including the superior edge of the SMH [38,39] and the area just below the submacular hemorrhage [34]. Sandhu et al. [40] suggested to perform from one to four retinotomies over the area of the submacular hemorrhage, away from the fovea [41]. In general, placing the retinotomy for the injection in an elevated retinal area can minimize the potential needle-related iatrogenic trauma. Okanouchi et al. [42] suggested to perform a limited ILM peeling in correspondence of the injection site with the aim to facilitate retinal penetration. Subretinal cannulas with size varying from 38- to 41-G are commonly used for the injection. Steel et al. [43] proposed filling the vitreous cavity with PFCL (approximately an 80% fill) before performing the subretinal injection in order to prevent foveal dehiscence. Indeed, the presence of PFCL ensures the gentle formation of a more diffuse bleb of reduced height and, thus, the minimization of the hydraulic stress on the foveal region during the injection [43].

The concentration of tPA used is more commonly 12.5 μg/0.1 mL [35], but can vary from 10 μg/0.1 mL [43] to 50 μg/0.1 mL [32]. The quantity injected can vary from 0.05 to 0.4 mL [5,38,39,41] to achieve a total dose ranging from 10 [41] to 50 μg [5,44]. Olivier et al. [34] suggested to encompass the whole hemorrhage with the therapeutic solution in order to create more space for the displacement of the blood. Some authors reported the use of 0.4 mL of rTPA at a concentration of 12.5 μg/0.1 mL combined with 0.1 mL of bevacizumab (2.5 mg) and/or 0.1–0.2 mL of filtered air [5,38]. In this case, when holding the syringe down, the air will float and locate far from the needle tip [5,38]. The subretinal injection of air can also be performed as a second step through the same retinotomy used for the initial tPA injection [39]. Adding air to the subretinal tPA is supposed to have several advantages, including contribution to the displacement of the blood downwards due to its direct pushing on the blood; protection of the macular area as the subretinal air is prevented to migrate further superiorly by the presence of the intravitreal gas; maintenance of the localized retinal detachment for few days, potentially allowing a prolonged action of the tPA and, thus, enhanced blood liquefaction; and increased oxygenation to the photoreceptors [45]. After the injection, an FAX is performed and different gases can be used as final intraocular tamponade, such as air [34,40], sulfur hexafluoride (SF6), or octafluoropropane (C3F8) [5,39,41].

Recently, the first in-human randomized controlled trial compared surgical outcomes (surgical success, operative time, and occurrence of any adverse event) of robot-assisted versus conventional manual subretinal injection of tPA for SMH [45]. This pilot study did not show any clinically significant difference between the two injection methods [45].

## 4. Subretinal Injection in Full-Thickness Macular Hole Repair

The subretinal injection of BSS to induce a macular detachment has been proposed as surgical option for the primary or, more commonly, secondary repair of FTMH [7]. The mechanism through which this technique could promote MH closure is the releasing of RPE–neuroretina adhesion and the consequent increased elasticity of the retina and, in particular, of hole edges that are more likely to become apposed [46]. In addition, the application of subretinal BSS may induce a plastic deformation of the retina, exceeding its yielding point and, thus, contribute to the reduction in diameter or closure of the hole [47,48]. Based on this rationale, the best candidates for this surgical technique could be the FTMHs that are likely to be characterized by firmer neuroretina–RPE adhesion, such as those associated with uveitis, trauma, or drusen, chronic, and/or refractory/recurrent FTMHs [46]. The reported rate of hole closure associated with this technique is >80% [7]; however, the visual gain may be lower than alternative surgical procedures [49]. In case of a secondary MH repair using this technique, positive prognostic factors may be a shorter intersurgical interval and smaller preoperative hole size [46,50], whereas sex, age, intraocular tamponade, and configuration of the hole may not have any influence on anatomical and functional outcomes [46,51].

### Surgical Technique

**Figure 3:**
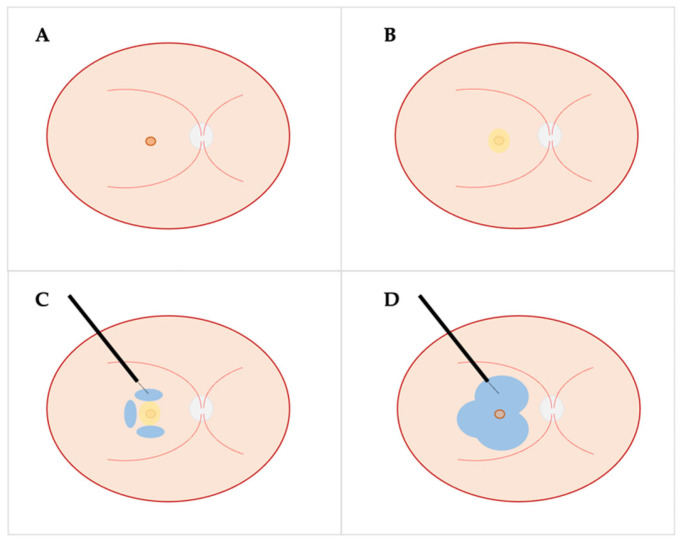
Subretinal injection for full-thickness macular hole: (**A**) schematic diagram of fundus with full-thickness macular hole; (**B**) injection of perfluorocarbon liquid until the edges of the hole are covered; (**C**) three subretinal blebs of BSS created using a subretinal cannula in different macular quadrants; (**D**) once PFCL is removed, the bubbles are further enlarged to involve the hole center.

Subretinal injection of BSS for FTMH repair is usually facilitated through standard 23- or 25-G PPV [52]. After PVD induction, core, and peripheral vitrectomy, vital dyes can be used to check the appropriate removal of vitreous and to facilitate ILM/ERM peeling; in case of previously vitrectomized eyes with refractory/recurrent FTMH, vital dyes can be injected to assess the extent of the pre-existing ILM peeling and the feasibility of an enlargement [7]. At this point, PFCL can be used to cover the hole edges and avoid the passage of BSS through the FTMH too early in the process (Figure 3B) [46]. The infusion pressure can be set lower than 20 mmHg to facilitate macular detachment, decreasing the resistance [46]. A 38/41-guage subretinal cannula is then used to create from two to four retinotomies, and corresponding BSS blebs, in different macular quadrants (Figure 3C). In particular, Meyer et al. [46] proposed to perform two retinotomies, one in the superior macula and the other in inferior macula, respectively, placed in the middle between the vascular arcades and the margins of the hole. As mentioned previously, subretinal BSS injection can be performed manually or using the foot pedal of the vitrectomy machine. Some authors suggested starting the injection of BSS slightly prior to retinal penetration in order to minimize potential iatrogenic retinal trauma [50]. Once all the planned BSS bubbles are formed, the PFCL can be removed and the bubbles are further enlarged to involve the hole center, ideally using the same retinotomy (Figure 3D). Multiple FAX can also contribute to expand the macular detachment and a centripetal massage of the detached retina can be performed prior to the last FAX [46,50,53]. Gurelik et al. [54] described a surgical variant in which the subretinal injection of a smaller amount of BSS is performed through a 39/41-gauge cannula around one disc diameter away from the hole temporally, superiorly, and inferiorly to create a localized detachment, involving the hole and the peri-hole area. After the passive aspiration of the hole edges using a silicone-tipped cannula, the same subretinal cannula is used to drain the subretinal BSS through the hole [54]. Fluorinated gases are usually preferred as final intraocular tamponade, but the use of silicone oil has also been described [7].

## 5. Complications

Subretinal injection can be associated with different intraoperative and postoperative complications; however, the exact rates of these complications have not been established due to the paucity of patients treated and of studies analyzing long-term outcomes.

Subretinal injection can cause iatrogenic RPE damage/irregularities due to direct mechanical trauma, both from the instrumentation and the flow rate of the therapeutic agent/BSS [7,16,20,21,55]. Mechanical trauma can also affect surrounding structures dependent upon where the cannula is placed. If the cannula is advanced too far, hydration or damage to the choroid, including hemorrhage, may occur, while a too-shallow placement can result in damage to retinal structures, including hydration of the outer retinal layers and retinoschisis [18]. Subretinal injection-related mechanical trauma has also been associated with the development of postoperative RPE atrophy appearing as an autofluorescent halo on fundus autofluorescence corresponding to the site of retinotomy [52] and the formation of choroidal neovascularization [56]. Furthermore, in the case of subretinal injection for gene therapy, an injection that is too deep can cause the break of the Bruch’s membrane and the potential egress of viral vector into the bloodstream [57]. In turn, this may result in choroidal granuloma formation or temporary hematic expression of gene products [57]. Conversely, in case of the subretinal injection of tPA, the risk of choroidal damage is reduced due to the presence of the blood clot.

Another severe complication that may occur is the formation of FTMH, usually as a result of overstretching of the fovea during the injection [15,16,17,20,21,25,55]. It is worth noting that, in case of subretinal injection for gene therapy, in addition to severely impacting the visual outcome, the iatrogenic formation of an FTMH can also cause the release of viral vector through the hole with the risk of loss of any therapeutic effect and intraocular inflammation [8,19,21].

Finally, as the subretinal injection is performed in the context of PPV, additional generic complications can be associated with the whole procedure, such as iatrogenic retinal tears, cataract formation, alterations of intraocular pressure, retinal detachment, endophthalmitis, bleeding, and vision loss [16].

### Subretinal Injection for Gene Therapy

There are a number of potential complications that are specifically associated with subretinal gene therapy injection. One of the main complications specific of subretinal gene therapy is the reflux of viral vector and the potential subsequent intraocular inflammation [12,14,15]. Indeed, the different viral vectors that are used may elicit differing inflammatory responses within the eye [12] As mentioned previously, this can be mitigated by the use of perioperative steroid therapy [12,15,16]. The enlargement of the retinotomy during the injection can increase the risk for reflux [16]. In this regard, the prolonged retention of the subretinal cannula within the retinotomy may minimize its enlargement and, thus, the reflux [26]. It has also been reported that the reflux might be associated with the postoperative development of ERM due to the intravitreal release of cells [58,59].

**Figure 4:**
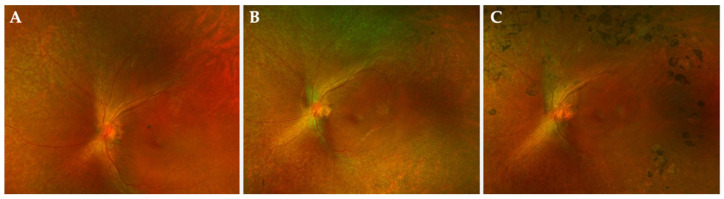
Atrophic changes after gene therapy. (**A**) Baseline; (**B**) 1 year after subretinal injection of voretigene neparvovec-rzyl, peripapillary area of chorioretinal atrophy; (**C**) 2 years after subretinal injection of voretigene neparvovec-rzyl, further enlargement of peripapillary chorioretinal atrophy.

Recently, the development of progressive perifoveal chorioretinal atrophy, not related to the site of injection, has been described as a postoperative complication of the subretinal injection of voretigene neparvovec-rzyl; however, no predisposing or intraoperative factors have been so far associated with this complication [60]. Atrophic changes with photoreceptor loss, but not significant impact on the maintenance of the functional results, have been recently reported both within the area of induced detachment and outside this area (Figure 4) [60]. However, photoreceptor loss just at the fovea has been reported, leading to reduction in visual acuity following subretinal injection of voretigene neparvovec-rzyl, presumably due to immune-related mechanisms [14].

## 6. Conclusions

Subretinal injection is now an established technique for drug delivery into the subretinal space, in particular due to the progressive evolution of gene therapy as well as, more recently, cell therapy. Indeed, ongoing trials are evaluating the therapeutic potential of therapies based on the delivery into the subretinal space of embryonal stem cells (hESCs) and human-induced pluripotent stem cells (hiPSCs) in eyes affected by age-related macular degeneration and Stargardt disease [61,62]. The huge advantage of a precise amount of drug delivery close to the potential target cells in the outer retinal layers and/or RPE outweighs the potential risks associated with this maneuver. Further studies are necessary to properly assess the potential impact of this technique on retinal and choroidal macro- and microstructure. In particular, the subretinal delivery of drugs through a suprachoroidal route via orbital delivery systems needs further exploration to avoid the need for vitrectomy and retinotomy. In the light of the expected growing application of subretinal injections, especially with the rapidly expanding field of retinal gene therapy for IRDs, a standardized and optimized surgical approach of subretinal injections appears to be of crucial importance for the effectiveness of the therapies whilst maintaining patient safety.

## Figures and Tables

**Table 1:** Inherited retinal diseases, target genes, and subretinal gene therapy assessed in ongoing clinical trials.

Inherited Retinal Disease	Gene Therapy	Targeted Gene	National Clinical Trial Number	Trial Phase
Achromatopsia	AAV2-mediated	CNGA3	NCT02935517	I/II
	AAV8-mediated	CNGA3	NCT02610582	I/II
	AAV2/8-mediated	CNGB3 or CNGA3	NCT03278873	I/II
LCA type 2	rAAV2-mediated	RPE 65	NCT00481546	I
	AAV2-mediated (voretigene neparvovec-rzyl) *	RPE 65	NCT01208389	I/II
			NCT00999609	III
			NCT04516369	III
			NCT03602820	IV
LCA type 10	AA5-mediated	CEP290	NCT03872479	I/II
Retinitis Pigmentosa	AA2-mediated	RPGR	NCT03316560	I/II
	AA2/5-mediated	hPDE6B	NCT03328130	I/II
	rAAV-mediated	hPDE6A	NCT04611503	I/II
	AAV8-mediated	RLBP1	NCT03374657	I/II
	AAV2-mediated (voretigene neparvovec-rzyl) *	RPE65	NCT00999609	III
			NCT04516369	III
			NCT03602820	IV

AAV, adeno-associated virus; EIAV, equine infectious anemia virus; LCA, Leber congenital amaurosis; rAAV, recombinant adeno-associated virus. * Approved by Food and Drug Administration in 2017 and by European Medicines Agency in 2018.

## Data Availability

Not applicable.
